# Dysregulation of Midbrain Dopamine System and the Pathophysiology of Schizophrenia

**DOI:** 10.3389/fpsyt.2020.00613

**Published:** 2020-06-30

**Authors:** Susan F. Sonnenschein, Felipe V. Gomes, Anthony A. Grace

**Affiliations:** ^1^Departments of Neuroscience, Psychiatry and Psychology, University of Pittsburgh, Pittsburgh, PA, United States; ^2^Department of Pharmacology, Ribeirao Preto Medical School, University of Sao Paulo, Ribeirao Preto, Brazil

**Keywords:** dopamine, ventral tegmental area, hippocampus, amygdala, thalamus, prefrontal cortex, medial septum

## Abstract

Dysregulation of the dopamine system is central to many models of the pathophysiology of psychosis in schizophrenia. However, emerging evidence suggests that this dysregulation is driven by the disruption of upstream circuits that provide afferent control of midbrain dopamine neurons. Furthermore, stress can profoundly disrupt this regulatory circuit, particularly when it is presented at critical vulnerable prepubertal time points. This review will discuss the dopamine system and the circuits that regulate it, focusing on the hippocampus, medial prefrontal cortex, thalamic nuclei, and medial septum, and the impact of stress. A greater understanding of the regulation of the dopamine system and its disruption in schizophrenia may provide a more complete neurobiological framework to interpret clinical findings and develop novel treatments.

## Introduction

Dopamine (DA) modulates circuit reactivity based on environmental stimuli and prior experience and thus plays a central role in functions including reward processing, reinforcement, and habit formation (1–3). Midbrain DA neurons have also been shown respond to novel or aversive stimuli in the absence of reward (4) and it has been proposed that DA signaling may more generally influence sensory processing, such as weighting the salience (5) or certainty (6) of perceived stimuli. Dysregulation of the DA system has been fundamental to many models of the pathophysiology of schizophrenia (7, 8). It is implicated particularly in psychotic symptoms, which involve profound perceptual disturbances (hallucinations) and fixed beliefs resistant to contradictory evidence (delusions). Hallucinations and delusions tend to co-occur and are thus proposed to manifest due to a common pathophysiological mechanism (6, 9). Psychotic symptoms can be attenuated by D_2_ receptor blocking drugs (10, 11) that reduce the abnormal increased DA neuron activity (12–14), but the underlying cognitive processes likely involve complex connections between numerous brain regions that remain dysfunctional. This article will discuss some of the circuits that regulate DA neuron activity and how dysfunction in these upstream circuits may influence the DA system in schizophrenia.

## Dopamine Dysfunction in Schizophrenia

Clinical imaging studies have provided strong support for the DA hypothesis of schizophrenia. Imaging studies that measured radioligand displacement from DA receptors as a measure of DA activity have shown that patients with schizophrenia display increased DA release in response to low-dose amphetamine, compared to healthy controls (15–17), which correlates with transient worsening of psychotic symptoms (17). Patients also demonstrate increased baseline levels of synaptic DA in the striatum, measured in a DA depletion paradigm (18), which has been shown to correlate with their amphetamine-induced DA release (19). Both measures are observed in antipsychotic drug-naive patients and drug-free patients with prior APD treatment, and both predict treatment response of psychosis to antipsychotic drugs (18–20). Elevated striatal DA synthesis capacity, measured by fluorodopa uptake into DA terminals, is also consistently observed in patients and shown to correlate with psychotic severity (21). Numerous studies have found increased response capacity of the DA system in individuals at clinical high risk (CHR) for psychosis, which correlates with greater severity of prodromal symptoms (22–25). Longitudinal studies have further shown that there is a progressive increase in striatal DA function as CHR patients transition to full syndrome expression (24), which has been shown to predict conversion to psychosis (23, 25). Elevated DA synthesis capacity is a less consistent finding in chronic patients in remission, shown to be significantly elevated compared to healthy controls in some studies (26–29), though not all (30–32), suggesting that increased DA function most clearly signals active psychosis. The elevation in DA is limited to striatal projections (33, 34). In contrast, mesocortical projections, particularly to the dorsolateral PFC, display reduced DA release compared to healthy controls, which may contribute to impaired prefrontal-dependent cognitive processes (35). It is currently unknown what accounts for these coexisting differences in DA regulation. Together, these findings point to dysregulation of the DA system as central to the development and expression of psychotic symptoms.

## Dopamine Neuron Projections to the Striatum

Midbrain DA neurons can be subdivided with respect to their location, projection target, and functional significance (36, 37). The striatum is one of the primary targets of DA signaling and receives dense projections from DA neurons following a topological gradient. In rodents, more medial DA neurons of the ventral tegmental area (VTA) innervate more reward-related ventral striatal regions, including the nucleus accumbens (38). More lateral DA neurons of the substantia nigra project to the dorsomedial and dorsolateral striatum, which are relevant to habit formation and motor function, respectively (39, 40). DA neurons that are located at the transition from lateral VTA to substantia nigra project to the rostral caudate, or associative striatum, which is most implicated in measures of increased presynaptic DA function and demonstrates the strongest correlation to psychotic symptoms in patients with schizophrenia (33, 34). In primates, the relative position of VTA DA neurons shift, but their topological organization is retained. Whereas rodents have a prominent VTA that is located medial to the substantia nigra, in the primate the DA neurons are shifted, with the rodent VTA projection to limbic and associative striatum now becoming the dorsal tier of the substantia nigra and the rodent substantia nigra that projects to the dorsal striatum now comprising the primate ventral tier substantia nigra neurons (41, 42).

## Activity States of Midbrain Dopamine Neurons

**Figure 1:**
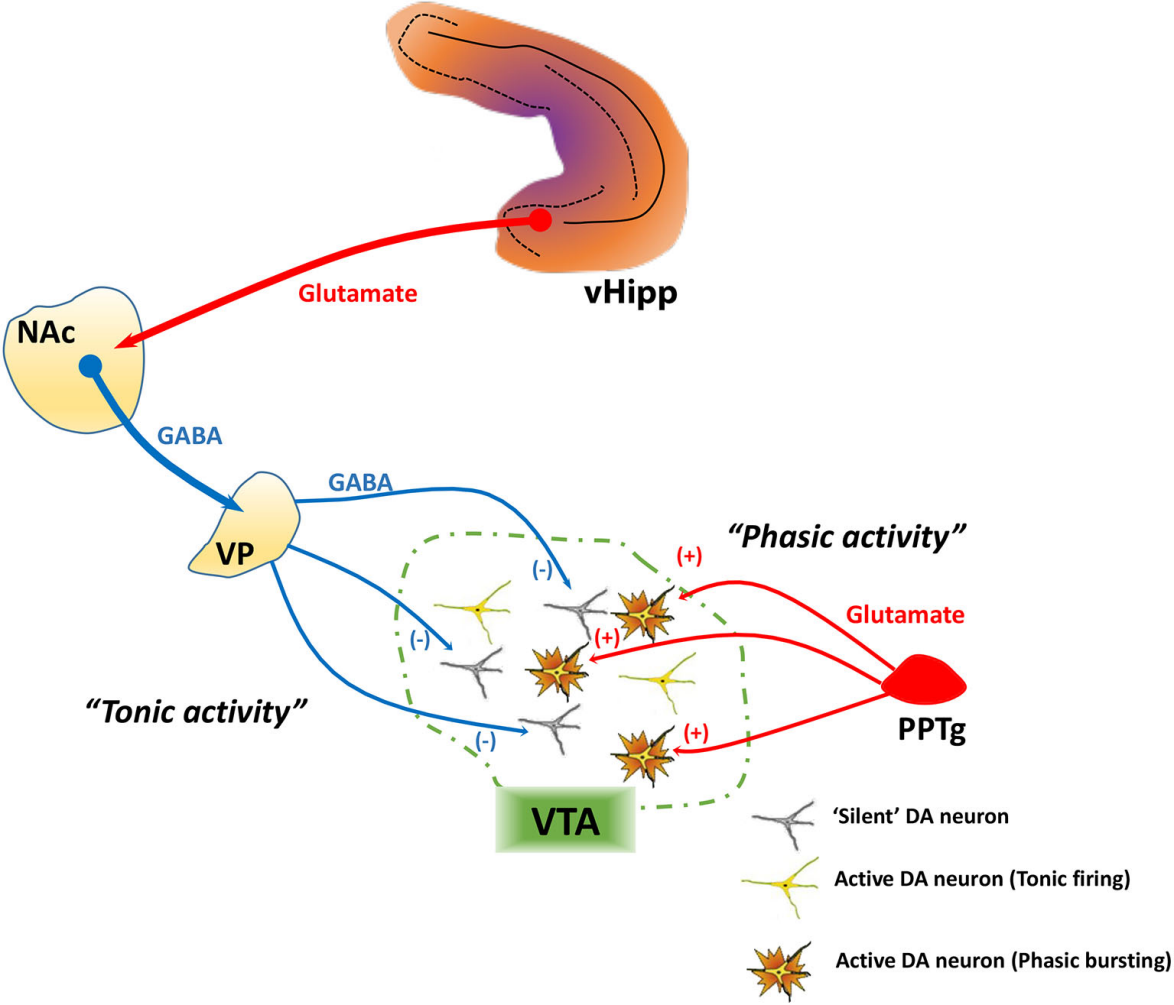
Tonic and phasic dopamine (DA) neuron activity are regulated by distinct afferent systems. DA neurons generate their own activity through a pacemaker conductance. However, a substantial population of DA neurons is not firing spontaneously, being held in a hyperpolarized state by a GABA-mediated inhibitory input from the ventral pallidum (VP). The VP, in turn, is controlled by a pathway originating from the ventral hippocampus (vHipp). The vHipp projects to the nucleus accumbens (NAc), which inhibits the VP. By contrast, phasic burst firing is driven by glutamatergic inputs arising from several areas, primary among these being the pedunculopontine tegmentum (PPTg). This afferent system regulates firing states within the population of spontaneously active DA neurons, because only neurons that are firing spontaneously can burst fire—NMDA channels on hyperpolarized (“silent”) DA neurons are under magnesium block and won't change state. Therefore, the PPTg provides the rapid, behaviorally salient phasic signal, whereas the VP, by controlling the number of DA neurons firing, determines the gain of the phasic signal.

DA neurons exhibit two patterns of activity, known as tonic and phasic states, that have different functional implications and are regulated by distinct afferent systems. In vitro in the absence of inputs, DA neurons maintain a basal activity state through the generation of a pacemaker conductance (43–45). However, in vivo recordings in normal rats have shown that not all DA neurons are showing spontaneous activity; instead, approximately half of midbrain DA neurons are not spontaneously active, and instead exist in a hyperpolarized state (45–47) due to inhibitory input from the ventral pallidum (48), an area that is regulated by a pathway that arises from the ventral subiculum of the hippocampus (vHipp). When the vHipp is activated, it provides a glutamatergic drive of GABAergic projection neurons in the nucleus accumbens, which in turn inhibits the ventral pallidum and increases the proportion of VTA DA neurons that are spontaneously active (i.e. “population activity”; **Figure 1**).

Spontaneously active DA neurons, in vivo, can display an irregular tonic firing pattern and rapid, phasic burst firing (45, 49, 50). Burst firing is dependent on glutamatergic afferents from the pedunculopontine tegmentum via activation of NMDA receptors (**Figure 1**) (48, 51). In DA neurons that are nonfiring, NMDA fails to activate NMDA receptors due to a magnesium block that is present at hyperpolarized membrane potentials (52). Thus, only DA neurons that are depolarized (spontaneously active) have the potential to exhibit burst firing. DA neurons exhibit burst firing when exposed to a behaviorally salient stimuli, such as a potential threat or reward (53, 54). Therefore, the number of neurons firing can control the amplitude of the behaviorally salient phasic burst response; when there are more DA neurons firing (i.e., greater population activity), NMDA will cause a greater number to exhibit phasic bursts, thus amplifying the phasic response to stimuli (45, 55). In other words, the vHipp-nucleus accumbens-ventral pallidum (vHipp-NAc-VP) circuit allows the baseline level of responsivity of the DA system, which is dependent on population activity, to be adjusted based on the context in which the stimuli are presented.

## Circuits That Influence VTA Dopamine Neuron Population Activity Through the vHipp-NAc-VP Pathway

Elevated DA system activity in schizophrenia results from dysfunction in a larger hippocampal-midbrain-striatal circuit, with a primary locus of pathophysiology that appears to develop in the vHipp. Deficits in the structure and function of the hippocampus are consistently observed in imaging and post-mortem studies of schizophrenia patients (56). Imaging studies show that the anterior hippocampus, which is homologous to the limbic vHipp in rodents (57), is hyperactive in individuals with schizophrenia (58). Most studies report increased hippocampal glutamate levels in both first-episode and chronic patients, independent of medication status (59), and changes in hippocampal metabolism and blood flow are associated with more severe psychotic symptoms in patients (60–62) and those at CHR (63, 64). Increased cerebral blood volume (CBV) has been reported specifically in the CA1 and subiculum of the hippocampus in patients with schizophrenia (65). Increased CBV is also present during the prodromal stage and predicts conversion to psychosis (66, 67) and hippocampal atrophy (68). Multiple lines of evidence have suggested that the hippocampal hypermetabolism is due to reduced parvalbumin (PV)+ GABA interneuron regulation of pyramidal neuron activity, secondary to excitotoxic degeneration of PV+ interneurons (69, 70). NMDA receptor antagonists, such as PCP and ketamine, may similarly exacerbate or mimic psychosis by blocking NMDA receptors on PV+ interneurons and thus disinhibiting pyramidal neurons (71, 72). This can lead to increased levels of glutamate and loss of PV+ interneurons following chronic NMDA receptor antagonist administration (68, 73–75).

One can induce an analogous disruption of hippocampal physiology in animal models based on developmental disruption, including the methylazoxymethanol acetate (MAM) neurodevelopmental rat model (76, 77). The MAM model involves administration of the mitotoxin MAM to pregnant dams on gestational day 17, which correlates with the vulnerable timepoint of the 2nd trimester in humans to adverse events such as maternal infection (78). The offspring of MAM-treated dams (“MAM rats”) develop region-specific disruption of neuronal maturation that results in adult phenotypes relevant to schizophrenia, in contrast to the offspring of dams that receive a saline injection, (“SAL rats”) (76, 79, 80). Adult MAM rats display loss of PV+ interneurons in the vHipp (81), resulting in a baseline hyperactive state from loss of inhibitory control of pyramidal cell activity (82). The increased vHipp drive results in an increase in DA neuron population activity through the vHipp-NAc-VP circuit and inactivation of vHipp in MAM rats can normalize the DA neuron activity and related aberrant behavior (48, 82). Taken together, these data suggest a that a loss of PV+ interneurons in the hippocampus leads to increased DA neuron population activity and a hyper-responsive DA state, in line with clinical evidence of increased presynaptic DA function (21, 42).

Several brain regions can enhance VTA DA system activity through interactions with the vHipp-NAc-VP pathway. Here we discuss evidence indicating the involvement of the medial prefrontal cortex (mPFC), thalamic nuclei, and medial septum on the VTA DA system and how changes in the activity of these regions may lead to a hyperdopaminergic state as seen in schizophrenia.

### Medial Prefrontal Cortex and the Regulation of the DA System

Dysfunction within the mPFC plays a central role in the pathophysiology of several psychiatric illnesses, including schizophrenia. For instance, contrary to the increased presynaptic striatal DA synthesis and release (83), it has been found that DA transmission is decreased in the PFC of schizophrenia patients (35). This cortical hypodopaminergic state is thought to be associated with impairments in cognitive and executive function in schizophrenia (35, 84). Also, a reduced PFC activity has been associated with elevated striatal DA function in schizophrenia patients and at-risk individuals (28, 85).

The mPFC is thought to be a major regulator of the DA system but with the outcome, either inhibitory and excitatory responses, reflecting the specific anatomy of mPFC afferents to the VTA. Two major mPFC subdivisions, the infralimbic (ilPFC) and the prelimbic (plPFC) cortices, send direct projections to the VTA (86) as well as to other regions linked with control of the midbrain DA system, such as the NAc (87). The ilPFC, in particular, seems to regulate the DA system activity through its modulation of the activity of the vHipp and basolateral amygdala (BLA). It was showed that the ilPFC exerts a bidirectional control over VTA DA system via the BLA and vHipp. Whereas the inactivation of the ilPFC increases VTA DA neuron population activity in a vHipp-dependent manner, the activation of the ilPFC decreased VTA DA neuron population activity (88). Compared with the ilPFC, the inactivation of the plPFC produced opposite effects on VTA DA neurons. Whereas the activation of the plPFC had no effect, the plPFC inactivation decreased VTA DA neuron population activity (88). This is consistent with the opposite manner that the ilPFC and plPFC impacts behavioral responses (89, 90). The mechanism by which the plPFC affects VTA DA system is still not completely understood, but it may involve the removal of plPFC attenuation of vHipp activity and/or removal of the inhibitory influence of the plPFC over the ilPFC.

Whereas vHipp activation upregulates DA responsivity, the amygdala decreases tonic DA neuron firing. Activation of the BLA has been shown decrease DA neuron population activity in the medial affect-related regions of the rat VTA, which is proposed to be due to a glutamatergic projection to the ventral pallidum, because blocking glutamate in the ventral pallidum prevents BLA activation-dependent down-regulation of DA neuron firing (91). Furthermore, the decrease in DA neuron population activity observed following activation of the ilPFC depends on an intact amygdala (88). Therefore, the opposing modulatory actions of the vHipp and the amygdala are determined by ilPFC activity.

It is worth noting that the mPFC does not project directly to the vHipp (92). Thus, the effect of ilPFC inactivation on increases in VTA DA neuron population activity, that was prevented by removal of the vHipp influence, may involve other brain regions such as the entorhinal cortex and thalamic nucleus reuniens (93, 94) since both receive direct excitatory projection from the ilPFC (92) and in turn provide powerful excitatory influence over the vHipp (92, 95, 96). Therefore, both the entorhinal cortex and nucleus reuniens could be a relay between the ilPFC and the vHipp that could potentially affect activity of DA neurons in the VTA.

### Thalamic Nuclei and Regulation of the DA System

The thalamus has long been implicated as a potential node of dysfunction in schizophrenia (97) mainly due to its heavily reciprocal connectivity with the hippocampus and prefrontal cortex (98–100), thus serving as a critical mediator of communication between these brain regions. Reductions in resting-state functional connectivity between the thalamus and the hippocampus and prefrontal cortex have been reported at both the chronic and early stages of schizophrenia. It has also been reported in at-risk individuals and may predict conversion to psychosis in this group (101–104). Thalamic dysconnectivity patterns consistent with those seen in schizophrenia were also observed in healthy individuals after receiving ketamine to model psychosis (105). Also, a reduction in sleep spindles, which are non-rapid eye movement sleep oscillations generated by the thalamic reticular nucleus, has been consistently reported in schizophrenia patients, and the magnitude of this reduction was inversely correlated with the severity of psychotic symptoms (106, 107). These findings suggest that the thalamus may serve as a hub of wide-scale network dysfunction in schizophrenia.

Recent rodent studies have similarly indicated circuit abnormalities underlying and resulting from thalamic dysfunction (108). The thalamus is composed of multiple nuclei, each with their distinct afferent and efferent projections (109). Our group has focused on the nucleus reuniens, a thalamic midline nucleus, since it is bidirectionally connected to the hippocampus and prefrontal cortex (98, 99, 110). The nucleus reuniens, in rodents, forms the primary route of communication between the prefrontal cortex and vHipp and is essential for behaviors involving coordinated action of these two regions, such as spatial navigation and fear memory (111–113). Regarding corticothalamic projection to nucleus reuniens, pyramidal neurons from layers 5 and 6 of the medial prefrontal cortex send direct projections to the nucleus reuniens (114, 115) and some neurons from layer 6 of the ilPFC neurons send collaterals to the antero-medial portion of the thalamic reticular nucleus (116), the same subregion of the thalamic reticular nucleus that projects to reuniens (117).

**Figure 2:**
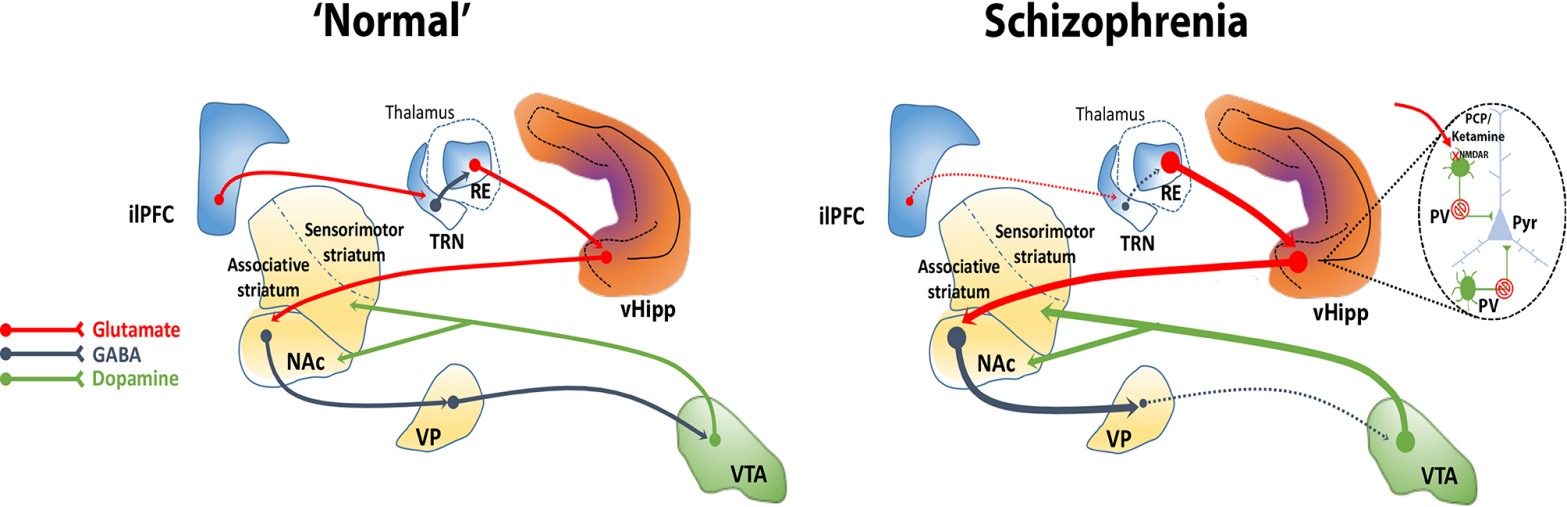
The ventral hippocampus (vHipp) regulates midbrain DA system activity through a polysynaptic circuit. The vHipp excites neurons in the nucleus accumbens (NAc) that, in turn, inhibit ventral pallidal (VP) activity. Given that the VP provides an inhibitory tone to VTA DA neurons, activation of the vHipp results in an enhance VTA DA neuron activity. In schizophrenia, a PV+ interneuron cell loss combined with a disruption of corticothalamic projections contributes to the hyperactivity of glutamatergic pyramidal (Pyr) neurons in the vHipp that drives an increase in active DA neurons projecting to the associative striatum that underlies the emergence of psychotic symptoms in schizophrenia. NMDA receptor antagonists, such as PCP and ketamine, may similarly exacerbate or mimic psychosis by blocking NMDA receptors on PV+ interneurons and thus disinhibiting Pyr neurons. Corticothalamic-hippocampal abnormal interactions can induce a hyperdopaminergic state, for instance, through a dysfunction of the medial prefrontal cortex (mPFC) that could disinhibit the nucleus reuniens (RE), possibly via loss of feedforward inhibition from the reticular nucleus of the thalamus (TRN).

We showed that activation of the nucleus reuniens increases DA neuron population activity in the VTA via its projection to the vHipp, since it was prevented by vHipp inactivation (93). Also, as described above, the inactivation of the ilPFC increases DA neuron population activity in the VTA, an effect that was dependent on the vHipp (88). The mPFC, however, does not send direct projections to the vHipp (92). Besides sending dense projections to the vHipp (96), the nucleus reuniens drives vHipp activity (118). The ilPFC inactivation enhances VTA DA system activity via vHipp likely by disinhibiting the nucleus reuniens since the inactivation of the nucleus reuniens prevented these changes (88). These findings suggest that 1) the ilPFC potently regulates the vHipp via nucleus reuniens and 2) the ilPFC inhibition leads to disinhibition of nucleus reuniens, likely due to deactivation of the thalamic reticular nucleus, which in turn, via its excitatory projections to the vHipp, enhances VTA DA system activity. The ilPFC was found to modulate several aspects of the firing pattern of neurons in the nucleus reuniens (119). Thus, the nucleus reuniens may mediate the regulation of the VTA DA system activity by the ilPFC. Overall, these findings suggest that a loss of top-down prefrontal regulation via disruption of corticothalamic communication, as has been observed in schizophrenia, could contribute to hippocampal overdrive and, consequently, to the hyperdopaminergic state characteristic of the disorder (**Figure 2**).

Another thalamic nucleus recently implicated in the regulation of the VTA DA system is the paraventricular nucleus of the thalamus. It was observed that the pharmacological activation of the paraventricular nucleus of the thalamus enhanced VTA DA neuron population activity, which was completely prevented by the inactivation of either vHipp or NAc (120). Both the paraventricular nucleus of the thalamus and vHipp send extensive glutamatergic innervation to the NAc (121, 122). Interestingly, the inactivation of the paraventricular nucleus of the thalamus attenuated the increased VTA DA neuron population activity induced by the vHipp activation (120). Moreover, this regulation seems to simultaneously require activity in both the vHipp and paraventricular nucleus of the thalamus. The inactivation of the paraventricular nucleus of the thalamus reverses vHipp-induced increases in VTA DA neuron population activity. Similarly, vHipp inactivation reverses the paraventricular nucleus of the thalamus-induced increases (120). Together, these findings suggest that convergent glutamatergic inputs from the vHipp and paraventricular nucleus of the thalamus to the NAc work in concert to regulate VTA DA neuron activity. In addition, the inactivation of the paraventricular nucleus of the thalamus reverses the abnormal increase in VTA DA neuron population activity exhibited by MAM rats (120), similar to what is observed after the inactivation of the vHipp in MAM rats (81). These findings indicate that aberrant thalamic activity may contribute substantially to the hyperdopaminergic state seen in schizophrenia.

### Medial Septum and the Regulation of the DA System

Another brain region that may influence vHipp activity and, in turn, regulate midbrain DA system activity is the medial septum. The medial septum sends dense cholinergic and GABAergic projections to several hippocampal regions (123, 124), including the vHipp (125). These projections are critical for hippocampal theta oscillation (126, 127), a major operational mode of the hippocampus, which is thought to be indicative of cognitive processing of environmental information (128).

The GABAergic projections from the medial septum synapse primarily on PV+ interneurons in the hippocampus (124, 126, 129), which is the interneuron subtype associated with the hippocampal hyperactivity and downstream hyperdopaminergic state present in schizophrenia (8, 42). On the other hand, the cholinergic projections provide slow depolarization of their target pyramidal neurons (126). Thus, the GABAergic and cholinergic projections from the medial septum can differently impact the excitatory-inhibitory balance in the vHipp which could ultimately lead to changes in the VTA DA system. In this context, our group recently found that pharmacological activation of the medial septum by a local infusion of NMDA increased the number of spontaneously active DA neurons in the VTA (130). An opposite effect was found in the substantia nigra. These effects induced by medial septum activation on both the VTA and substantia nigra depend on the vHipp since they were prevented by the inactivation of this brain region (130). Moreover, the effects of medial septum activation on VTA DA neuron population activity were also prevented by the infusion of the muscarinic receptor antagonist scopolamine into the vHipp, suggesting that medial septum cholinergic inputs to the vHipp may be involved in these effects (130). In addition, the inactivation of the anterior portion of the VP blocked the increased VTA DA neuron population activity induced by medial septum activation (130). On the other hand, inactivation of the posterior portion of the VP blocked the suppression of substantia nigra DA neuron population activity by medial septum activation. This suggests that there are topographically organized parallel circuits by which medial septum activity can bi-directionally affect DA neurons. Also, these findings indicate that medial septum seems to modulate midbrain DA system activity via the vHipp-NAc-VP pathway.

These opposite actions on VTA and substantia nigra DA neurons mediated by medial septum activation were recently associated with an enhancement of cognitive flexibility (131), a process profoundly attenuated in schizophrenia (132). The concept is that activation of the VTA causes the subject to think about the action, while attenuation of the substantia nigra prevents action until after weighing options. Interestingly, the regulation of the midbrain DA system activity by the medial septum in the MAM model of schizophrenia is different from that observed in normal rats (133). Whereas medial septum activation increases VTA DA neuron population activity and inhibits the substantia nigra in the normal rat (130, 133), an activation of the substantia nigra and a reduction of the abnormal increased VTA DA neuron population activity in MAM rats to baseline levels was observed (133). A possible explanation for these findings is that, in MAM rats, medial septum activation leads to an increase in the pyramidal neuron inhibition which would mitigate the vHipp hyperactivity (81). For example, the medial septum activation in normal rats leads to the release of GABA from the medial septum GABAergic projections to vHipp (123, 125). Since interneurons tend to be more sensitive to GABA than pyramidal neurons (134, 135), the released GABA would activate GABA_A_ receptors on interneurons in the vHipp. This would inhibit interneurons, which in turn leads to the disinhibition of pyramidal neurons. On the other hand, in MAM rats, GABA released in the vHipp induced by the medial septum activation would be more likely to reach pyramidal neurons due to the loss of interneurons in the vHipp. These changes combined with the loss of cholinergic activation of a parallel set of GABAergic projections to vHipp pyramidal neurons that impact the substantia nigra (123, 124) would increase vHipp drive of the VTA while increasing vHipp inhibition of the substantia nigra. Therefore, in contrast to the control condition, in the MAM rats the excitation of the substantia nigra combined with inhibition of the VTA would cause the subject to act before thinking, or causing impulsive behavior (133). Overall, the findings observed in MAM rats indicate that the medial septum-vHipp pathway as a potential target to reverse the hyperdopaminergic state in schizophrenia patients.

## Impact of Stress on VTA Dopamine Neuron Regulation

A diathesis-stress model proposes that schizophrenia develops due to stress exposure acting on a pre-existing vulnerability (136). Indeed, a large body of work highlights the importance of stress as a risk factor in the development of schizophrenia (43, 137–139). Early life stress and chronic social stressors, in particular, have been shown to increase the risk of schizophrenia (140, 141). Acute stress can trigger psychotic symptoms (142) and impaired stress tolerance is associated with prodromal symptoms (143). The correlation between early life stress and severity of positive symptoms (144) may partially be due to the interaction between stress, the hippocampus, and the DA system (145, 146).

The vHipp, which is integral in regulating context-dependent responses (147, 148), also shows marked vulnerability to stress across many psychiatric conditions. This may in part be due to a high expression of glucocorticoid receptors to respond to activation of the hypothalamic-pituitary-adrenal (HPA) axis (149). While an elevation in glucocorticoids is essential to respond to perceived threat, chronic elevation can result in impaired function and hippocampal atrophy (150, 151). This would be exacerbated by stress-induced activation of amygdala-hippocampal glutamatergic projections that target PV+ interneurons (152). Prolonged stressors can lead to dendritic shrinkage and neuronal loss in the hippocampus (149), including a loss of PV+ interneurons (153). It has thus been hypothesized that vHipp dysfunction may contribute to the diathesis in prodromal patients that puts them at risk for developing psychosis in response to stress (154).

Both CHR individuals and schizophrenia patients demonstrate elevated DA release in response to stress compared to healthy controls (155, 156). In adult rats, prolonged stressors, such as restraint stress (157) or repeated footshock (158), increase DA neuron population activity and the level of DA in nucleus accumbens (159). The increase in DA neuron activity can be normalized by inhibiting the vHipp (157, 158). However, at later timepoints, there is a compensatory reduction in DA neuron population activity, referred to as an opponent process (160), and shown to be dependent on the BLA (146, 161). In contrast, during puberty, prolonged stress exposure in rats has been shown to result in a long lasting increase DA neuron activity in adulthood, suggesting that stress before or during puberty is particularly impactful to the responsivity of the DA system (162, 163).

Heightened stress responsivity, insufficient prefrontal inhibition activity in the amygdala (152, 164, 165), and general loss of corticothalamic communication, may contribute to vHipp dysfunction and the emergent hyperdopaminergic state. Extreme stress, or a failure of the PFC to mitigate the impact of stress, could lead to loss of PV+ interneurons in the hippocampus in late adolescence or early adulthood. This in turn would lead to hippocampal hyperactivity and DA system dysregulation. We have shown previously that peripubertal administration of the benzodiazepine diazepam, can prevent the increased anxiety-like behavior and BLA hyperactivity, and normalize hyperdopaminergic activity typically present in adult MAM rats (166–168). These studies suggest that increased stress responsivity, particularly at crucial developmental stages, could lead to the emergence of psychosis in adults and that decreasing stress or other means of reducing vHipp activity during peripubertal period has the potential to circumvent the pathological processes that leads to DA system dysregulation (8). Evidence from animal studies indicate that sex differences should be taken into account since female rodents appear to show greater resilience to schizophrenia-like traits resulting from developmental stress (169). These findings may be associated with the delayed onset and lesser severity of schizophrenia in females (170, 171).

## Conclusion

The DA system has long been implicated in the expression and treatment of psychotic symptoms in schizophrenia. Study of the circuits that drive DA dysfunction can provide greater and more integrative understanding of a system-wide pathophysiology. Disruption of these circuits through developmental insults and pathological stressors can lead to DA system dysregulation. Ultimately, a greater understanding of the circuits that drive DA system dysfunction in schizophrenia can provide a neurobiological basis for interpreting clinical studies and potential targets for the treatment and prevention of schizophrenia and related psychotic disorders.

## Author Contributions

All authors contributed to the article and approved the submitted version.

## Funding

Research activity of the authors is supported by grants from US National Institutes of Health (MH57440 to AG) and Sao Paulo Research Foundation (FAPESP Young Investigators Grant - 2018/17597-3 to FG).

## Conflict of Interest

AAG has received consulting fees from Alkermes, Lundbeck, Takeda, Roche, Lyra, Concert, and research funding from Lundbeck.

The remaining authors declare that the research was conducted in the absence of any commercial or financial relationships that could be construed as a potential conflict of interest.
